# Evaluation of Anti-plaque and Anti-gingivitis Efficacy of Two Commercially Available Herbal and Non-herbal Toothpastes

**DOI:** 10.7759/cureus.39558

**Published:** 2023-05-27

**Authors:** Yogesh Garg, Zoya Chowdhary, Kamal Garg, Minal M Kshirsagar, Arpit Sharma, Jaidupally Ramvilas Reddy, Kapil Paiwal

**Affiliations:** 1 Public Health Dentistry, Jan Nayak Chaudhry Devi Lal (JCD) Dental College, Sirsa, IND; 2 Periodontology, Indira Gandhi Government Dental College, Jammu, IND; 3 Periodontology, Institute of Technology & Science (ITS) Dental College, Greater Noida, IND; 4 Public Health Dentistry, Nair Hospital & Dental College, Mumbai, IND; 5 Public Health Dentistry, Daswani Dental College & Research Center, Kota, IND; 6 Public Health Dentistry, Kliniq32 Dental Implant & Cosmetic Centre, Shankarpally, IND; 7 Oral & Maxillofacial Pathology, Daswani Dental College & Research Center, Kota, IND

**Keywords:** non-herbal, herbal, dental plaque, dentifrice, gingivitis

## Abstract

Background: Plaque-associated oral disease affects a considerable portion of the population and is considered one of the major causes of tooth loss. The presence of plaque may be the reason for dental caries, gingivitis, periodontal problems, and halitosis. Many mechanical aids are used to control plaque, including toothbrushes, dental floss, mouth rinses, and dentifrices, and the most effective method of controlling gingivitis is supragingival plaque control.

Aim and objective: To evaluate and compare the anti-plaque and anti-gingivitis efficacy of commercially available herbal toothpaste (Meswak) and non-herbal toothpaste (Pepsodent).

Materials and method: 50 subjects aged between 10 and 15 years with a full complement of dentition were included in the study. The two toothpastes were provided to the subjects in plain white tubes by the investigator. Subjects were instructed to brush their teeth twice daily using the given toothpaste for 21 days. Plaque and gingival scores on days 0, 7, and 21 were recorded, and the data were subjected to statistical analysis.

Result: At the end of the 21-day study, there was a statistically significant difference between the groups for plaque and gingival scores.

Conclusion: The plaque and gingival scores were significantly reduced throughout the study in both groups. In comparison, the herbal dentifrices show more effectiveness in reducing plaque and gingival scores, but no statistically significant difference was seen between the two groups.

## Introduction

Dental plaque may be defined as a specific but highly variable structural entity resulting from the colonization and growth of microorganisms on the surfaces of teeth and consisting of numerous microbial species and strains embedded in an extracellular matrix. Clinically, it occurs in the supragingival and subgingival areas and may also be found on other solid surfaces, such as restorations and oral appliances [1]. The precursor for dental caries, gingivitis, and periodontitis is plaque. For the prevention and control of these precursors, we need to have optimal plaque control [2].

In India, >50% of the population is affected by dental caries and periodontal disease, which in turn significantly affect general well-being and overall quality of life [3]. The importance of regular oral hygiene practices has been emphasized by the European Workshop of 1998 [4]. Since then, researchers have focused on in-vitro experiments, clinical trials, and demonstration projects in different geographical regions and social settings to confirm the effective removal of dental plaque, which is essential to dental as well as periodontal health [5]. Natural and herbal products, with their rising popularity, have mandated professionals to evaluate their effectiveness to provide better choices for patients on evidence-based suggestions [5].

Oral hygiene includes any or all of the procedures that contribute to a state of good oral health. To achieve improved oral health, many techniques and products are available nowadays, such as toothbrushes, rinses, floss, and dentifrices [3]. The commonly used dentifrices are synthetic and contain chemical agents that are known to produce harmful side effects with prolonged use [6].

Chlorhexidine is one of the most effective supragingival plaque control agents. However, in a toothpaste preparation, a significant reduction of its antiplaque potential may be observed [7]. Triclosan has also shown a significant reduction in plaque and gingivitis [8]. Additional research is required for antimicrobial dentifrices that are comparatively less abrasive in controlling bacterial plaque. Controlled clinical trials have demonstrated that brushing teeth with herbal dentifrices reduces supragingival plaque as well as gingivitis [9,10].

Herbal dentifrices have several benefits due to their ingredients: chamomile for its anti-inflammatory effect; echinacea for its immune stimulatory effect; sage and rhatany for their anti-hemorrhagic properties; myrrh is a natural antiseptic; and peppermint oil for its analgesic, antiseptic, and anti-inflammatory properties [10,11].

"Natural" dentifrices typically do not include ingredients such as synthetic sweeteners, artificial colors, preservatives, additives, synthetic flavors, and fragrances. They are formulated from naturally derived components. For example, in "natural" toothpaste, the fluoride comes from fluorspar, the abrasive system is calcium carbonate (chalk) in place of synthesized abrasive, the thickener is carageenan (derived from seaweed), and the sweetener is xylitol (a product extracted from birch trees) instead of saccharin [12].

Recent studies have shown that herbal extracts have significant antimicrobial effects against plaque-forming bacteria, as a result of which various herbal agents are introduced in dentifrices and mouth rinses [11,13]. But for most people, brushing alone is inadequate to remove plaque. However, several active antimicrobial agents are incorporated to prevent the microbial disease.

In addition to antimicrobial activity, certain medicinal plants/herbs have multi-potential therapeutic effects that, if incorporated into dentifrices, may be effective for long-term use. Researchers have been investigating chemical agents that could reduce/prevent oral disease. Thus, an attempt was made to evaluate and compare the anti-plaque and anti-gingivitis efficacy of commercially available herbal and non-herbal toothpaste among school-going children.

## Materials and methods

A double-blinded randomized clinical trial was conducted to evaluate and compare the anti-plaque and anti-gingivitis efficacy of commercially available herbal and non-herbal toothpaste.

Inclusion and exclusion criteria

The inclusion criteria were as follows: subjects with a plaque index of >1 (as measured by the Quigley-Hein plaque index modified by Turesky et al., 1970) [14]; subjects with a gingival index >1 (as measured by Loe (&) Silness, 1963) [15]; both male and female children aged between 10 and 15 years; systemically healthy individuals; participants willing to give consent. Those who were not willing to sign the consent form, and those suffering from medically compromised conditions were not included in the study.

Study design

A randomized clinical trial which included 50 subjects, both male and female, was conducted to evaluate and compare the efficacy of two commercially available herbal and non-herbal toothpaste. The ethical clearance was obtained from the Institutional Ethical Committee of Teerthanker Mahaveer University (approval no. TMU/EC/337). Written informed consent was obtained from participants' parents/ guardians/attendees before commencing the study, which was in accordance with the World Medical Association’s Declaration of Helsinki [16]. Subjects in each group were shown a demonstration of brushing techniques using the cast model i.e., the Fones technique (by placing the brush over a set of teeth, not at a particular angle, and then brushing each set four to five times with gentle, circular motions. Subjects were later asked to brush their teeth using the technique demonstrated to them in front of a mirror, which was supervised. 

Impact of brushing technique

Toothbrushing is considered fundamental self-care behavior for the maintenance of oral health, and brushing twice a day has become a social norm, but the evidence base for this frequency is weak. It seems to be effective in reducing mean periodontal disease and mean calculus progressions, besides increasing the number of teeth retained. The modified Bass technique is the most effective brushing technique, but due to a lack of brushing skill and the required manual dexterity for tooth brushing, the Fones method of toothbrushing was preferred.

Method of data collection

The 50 subjects were randomly selected. The subjects were randomly divided into two groups: group 1 (herbal) and group 2 (non-herbal), with a flip of a coin. The study was conducted over 21 days.

Prior to the commencement of the study, subjects underwent hand scaling to remove plaque, stain, and calculus from the tooth surface, by using a disclosing agent. All the subjects underwent a washout period of 2 ½ days to rule out any possible carry-over effects of the antecedent pre-owned oral hygiene products. The washout period was followed by a treatment period of 21 days.

The plaque and gingival index scores were recorded at baseline i.e., on day 0 of all the subjects. The subjects were then provided with plain white tubes containing toothpaste and the subjects were unaware of which group they were in.

Group 1 subjects received commercially available herbal dentifrice (Meswak) inclusive of 1000 ppm sodium monofluorophosphate, calcium carbonate, chamomile, eucalyptus, myrrh, and sage. Group 2 subjects received conventional dental cream (Pepsodent) containing 1000 ppm sodium monofluorophosphate, calcium carbonate, silica, and triclosan. The plaque and gingival index scores were recorded on days 0, 7, and 21.

Statistical analysis

The collected data were statistically analyzed by SPSS Statistics version 20 (IBM Corp., Armonk, NY, USA). Independent t-test, paired sample t-test, and repeated measure ANOVA were used to find out the comparisons between the two groups. The p-values ≤0.05 were considered statistically significant.

## Results

Fifty randomly selected subjects were included in the study and randomly divided into two groups: group 1 (herbal) and group 2 (non-herbal), to evaluate and compare the anti-plaque and anti-gingivitis efficacy of commercially available herbal and non-herbal toothpaste. Intergroup comparisons showed a statistically significant difference in the reduction of plaque using repeated measure ANOVA from day 0 but after day 21, group 1 showed an average 62.57% reduction and group 2 showed a 61.53% reduction. On the other hand, the independent t-test showed no statistically significant difference on days 7 and 21. It was also seen that the highest percentage reduction of plaque was achieved on the buccal surface: 32.78% and 31.21% in groups 1 and 2, respectively (Table 1).

**Table 1:** Comparison of herbal and non-herbal toothpaste based on overall plaque index *t: Independent t-Test; F: Value of repeated measure ANOVA; p-value: Level of significance where <0.05 is statistically significant and >0.05 is statistically non-significant

Selected teeth	Examination day	Group 1 (herbal)		Group 2 (non-herbal)			
		Mean	SD	Mean	SD	t-value	p-value
All	0	1.71	0.14	1.69	0.20	0.619	0.544
	7	1.20	0.18	1.36	0.12	1.049	0.990
	21	0.64	0.07	0.65	0.11853	0.013	0.338
		F-value= 35.65, p-value<0.001, %Reduction=62.57%		F-value=32.45, p-value<0.001, %Reduction=61.53%			

**Figure 1:**
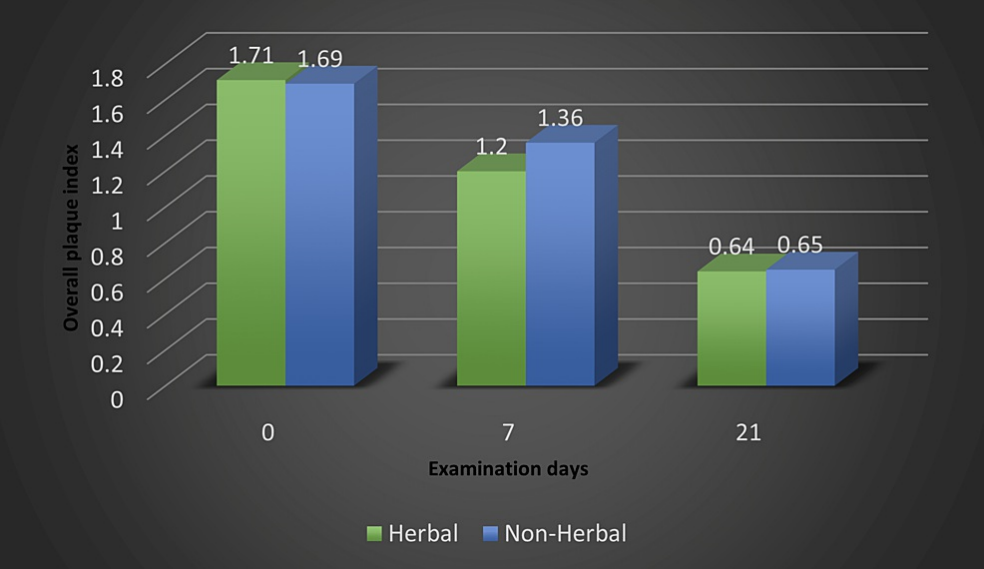
Comparison of herbal and non-herbal toothpaste based on overall plaque index

Figure 1 shows the comparison of the overall plaque index of both herbal (group 1) and non-herbal (group 2) toothpaste. The mean for group 1 for days 0, 7, and 21 were 1.71+0.14, 1.20+0.18, 0.60+0.07; and for group 2 were 1.69+0.20, 1.36+0.12, 0.65+0.11853, respectively (Figure 1). 

The overall mean for the maxillary arch of group 1 on days 0, 7, and 21 were 1.75+0.54, 1.24+0.62, and 0.50+0.44 whereas; for the mandibular arch it was 1.61+0.32, 1.15+0.44, 0.83+0.43, respectively. On the other hand, the overall mean for maxillary and mandibular arches of group 2 were 1.73+0.43, 1.49+0.54, 0.63+0.33 and 1.60+0.65, 1.26+0.54, 0.78+0.32. On comparison of the mean plaque scores for the maxillary arch using repeated ANOVA showed that a significantly low plaque score was obtained at day 21 in both groups, whereas in the mandibular arch, the mean plaque score was low for both groups on day 7 and day 21 when compared to day 0. In both the arches, plaque scores were lower in group 1 only on day 7, and on the follow-up days, a non-significant difference was observed in both groups. It also depicts that the maxillary arch achieved the highest percentage reduction (71.42% and 63.58%), followed by the mandibular arch (48.44% and 51.25%) in both groups, respectively. A better reduction in the maxillary arch was observed in the herbal group whereas more reduction in the mandibular arch after day 7 was observed in the non-herbal group (Table 2).

**Table 2:** Comparison of herbal and non herbal toothpaste based on arch-wise plaque index *t: Independent t-Test; F: Value of repeated measure ANOVA; p-value: Level of significance where  <0.05 is statistically significant and >0.05 is statistically non-significant

Selected teeth	Examination day	Group 1 (herbal)		Group 2 (non-herbal)			
		Mean	SD	Mean	SD	t-value	p-value
Maxillary	0	1.75	0.54	1.73	0.43	0.98	0.90
	7	1.24	0.62	1.49	0.54	1.21	0.52
	21	0.50	0.44	0.63	0.33	1.04	0.60
		F-value=36.48, p-value<0.001, %Reduction=71.42%		F-value=34.31, p-value<0.001, %Reduction=63.58%			
Mandibular	0	1.61	0.32	1.60	0.65	0.66	0.54
	7	1.15	0.44	1.26	0.54	0.76	0.43
	21	0.83	0.43	0.78	0.32	0.84	0.44
		F-value=32.45, p-value<0.001, %Reduction=48.44%		F-value=31.44, p-value<0.001, %Reduction=51.25%			

However, the difference was not statistically significant between the groups. Intergroup comparisons of both the groups using repeated measure ANOVA of mean gingival scores demonstrated statistically significant differences throughout the study. However, on day 21, 53.01% reduction in the herbal (group 1) group and a 40.0% reduction in the non-herbal (group 2) group was seen. There was no statistically significant difference observed among any of the recorded days on comparison between mean gingival scores of both groups using an independent t-test, as shown in Table 3.

**Table 3:** Comparison of herbal and non-herbal toothpaste based on overall gingival index *t: Independent t-Test; F: Value of repeated measure ANOVA; p-value: Level of significance where  <0.05 is statistically significant and >0.05 is statistically non-significant

Selected teeth	Examination day	Group 1 (herbal)		Group 2 (non-herbal)			
		Mean	SD	Mean	SD	t-value	p-value
All	0	0.83	0.22	0.75	0.20	0.62	0.54
	7	0.65	0.13	0.64	0.12	1.03	0.69
	21	0.39	0.21	0.45	0.16	0.01	0.43
		F-value=38.65, p-value<0.001, %Reduction=53.01%		F-value=32.35, p-value<0.001, %Reduction=40.0%			

**Figure 2:**
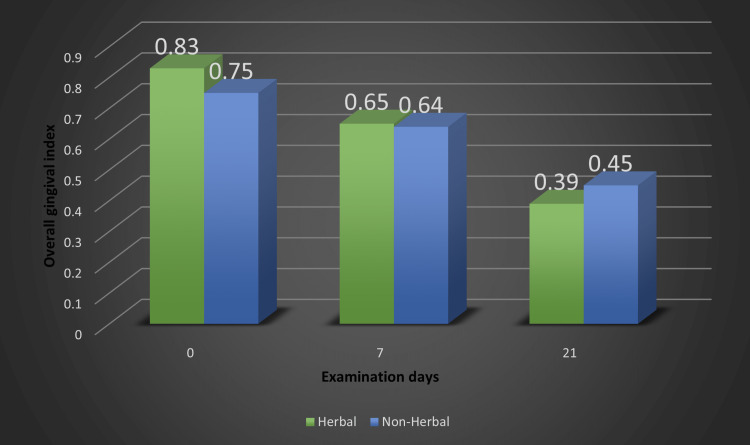
Comparison of herbal and non-herbal toothpaste based on the overall gingival index

Figure 2 shows the overall comparison of gingival indices in the herbal and non-herbal groups. The mean for group 1 on days 0, 7, and 21 were 0.83+0.22, 0.65+0.13, 0.39+0.21, respectively, and 0.75+0.20, 0.64+0.12, 0.45+0.16, respectively, for group 2 (Figure 2). 

## Discussion

The present study shows the comparison between herbal and non-herbal dentifrice in children. Herbal dentifrices have several medicinal properties such as natural antiseptics that tend to kill dangerous micro-organisms in the mouth, tannic acid's astringent property which is useful for the gums, and organic oils that improve salivation [13,17]. They also contain fluorides that help maintain oral health by protecting the tooth enamel layer, and also the color of the tooth [18]. "Meswak contains ‘silica’, which in turn helps teeth bleach" [19]. Other ingredients in Meswak such as fluorides are helpful as a structural element of pungent skeletal system and teeth; silicon is indispensable for the repair of connective tissue, cartilage, bone tissue, hair, nails, teeth, and calcium; and sulfate is essential in the growth of teeth and bones [19]. The gypsum crystals employed in Meswak act as cleaning agents. They assist to resolve the adherent particles from the teeth. Other components are tannins, saponins, vitamin C, flavonoids, and chlorides [19].

Dental caries can occur in the presence of dental plaque. Hence, the antibacterial efficacy of dentifrices is one of the key factors in selection of the toothpaste. The ingredients present in toothpaste kill microbes and reduce their growth and colonization on the tooth surface [5]. With an increased interest in natural-based toothpaste, herbal extracts have gained special attention as they are non-chemical and non-synthetic, and are also being used in traditional medicine [12]. Studies have shown that the use of herbal dentifrices reduces dental plaque accumulation on tooth surfaces as well as in interproximal areas probably because of the active ingredients in herbal dentifrices [1,3,9-11,13].

Triclosan is an antimicrobial agent with well-established safety and efficacy [8]. Fluoride has an anti-caries effect, but some of the constituents of toothpaste have unacceptable side effects such as staining and taste alterations [20]. Hence, natural products with added benefits are advised for use. Several studies have proven the anti-plaque and anti-gingival effects of herbal toothpaste, which were comparable to those of conventional toothpaste [1,3,9-11,13,19].

In the present study, both groups showed a highly significant (p<0.001) difference in mean dental plaque scores for both maxillary and mandibular arches throughout the study period. A statistically significant mean gingival bleeding score was seen in both groups between day 0 and day 21. However, the differences were not statistically significant between the groups for both dental plaque and gingival bleeding. The reduction in plaque in both groups was seen in a week which could be attributed to supervised brushing and the fluoride content in the dentifrice [2]. The findings are in accordance with the studies by Saxer et al. [21] and Mullaly et al. [22].

The results of this study also demonstrated that gingival inflammation and bleeding were significantly reduced in both groups in the first week. No significant difference was observed between both groups but, in the herbal group, a considerable reduction in both gingival inflammation and bleeding was seen. It could be because of the herbal dentifrice ingredients which have more anti-inflammatory and astringent properties than a non-herbal dentifrice [2]. Therefore, a reduction in dental plaque and restoration of gingival health may be obtained in a week under supervised tooth brushing.

Suresh et al. in his study observed that both herbal toothpaste and non-herbal toothpaste are equally powerful but no longer extra superior to fluoride toothpaste [23]. Nivethaprashanthi et al.'s study suggests that there is an increased interest in using herbal products [24]. Herbal toothpastes are as effective as non-herbal toothpastes in controlling plaque and gingivitis. There are also no adverse reactions to using herbal toothpaste and can be used as an alternative to conventional toothpaste. Both studies are in accordance with the present study.

Limitations

In the present study, two commercially available toothpastes are compared. However, the current market is filled with different kinds of herbal toothpastes. So, further studies should be undertaken to evaluate the effects of other toothpastes. The current study was undertaken for 21 days only; further long-term prospective studies should be undertaken to evaluate the effects of these toothpastes on oral hygiene. The sample size in this present study is very small, so a larger sample size should is required to obtain reliable and significant results.

## Conclusions

Herbal dentifrices restore gingival health and reduce plaque formation more effectively than non-herbal dentifrices. The overall results suggest that herbal dentifrices are an effective alternative to non-herbal dentifrices and are safer as they include natural ingredients. Nowadays, there is an increased interest in herbal products among people. Therefore, within the limits of this present study, it can be concluded that herbal dentifrices are safe, have no adverse effects, and are more effective than non-herbal (conventional) dentifrices in controlling plaque and gingivitis.
